# A Japanese boy with Bardet-Biedl syndrome caused by a novel homozygous variant in the *ARL6* gene who was initially diagnosed with retinitis punctata albescens: A case report

**DOI:** 10.1097/MD.0000000000032161

**Published:** 2022-12-16

**Authors:** Keitaro Mizumoto, Kumiko Kato, Kaoru Fujinami, Tadasu Sugita, Iichiro Sugita, Ayako Hattori, Shinji Saitoh, Shinji Ueno, Kazushige Tsunoda, Takeshi Iwata, Mineo Kondo

**Affiliations:** a Department of Ophthalmology, Mie University Graduate School of Medicine, Tsu, Japan; b Laboratory of Visual Physiology, Division of Vision Research, National Institute of Sensory Organs, National Hospital Organization Tokyo Medical Center, Tokyo, Japan; c UCL Institute of Ophthalmology, University College London, London, United Kingdom; d Department of Ophthalmology, Sugita Eye Hospital, Nagoya, Japan; e Department of Pediatrics and Neonatology, Graduate School of Medical Sciences, Nagoya City University, Nagoya, Japan; f Department of Ophthalmology, Hirosaki University Graduate School of Medicine, Hirosaki, Japan; g Division of Vision Research, National Institute of Sensory Organs, National Hospital Organization Tokyo Medical Center, Tokyo, Japan; h Division of Molecular and Cellular Biology, National Institute of Sensory Organs, National Hospital Organization Tokyo Medical Center, Tokyo, Japan.

**Keywords:** *ARL*6, Bardet-Biedl syndrome, BBS3, polydactyly, retinitis pigmentosa, retinitis punctata albescens

## Abstract

Bardet-Biedl Syndrome (BBS) is an autosomal recessive systemic disorder characterized by retinitis pigmentosa, polydactyly, obesity, intellectual disability, renal impairments, and hypogonadism. The purpose of this study was to determine the ocular characteristics of a boy with BBS caused by a novel homozygous variant in the *ARL6* (alternative named *BBS3*) gene who had been originally diagnosed with retinitis punctata albescens.

**Methods:**

This was an observational case study. The patient underwent ophthalmological examinations, electroretinography, and genetic analyses using whole-exome sequencing.

**Results:**

A 7-year-old boy was examined in our hospital with complaints of a progressive reduction of his visual acuity and night blindness in both eyes. There was no family history of eye diseases and no consanguineous marriage. Fundus examinations showed numerous white spots in the deep retina and retinal pigment epithelium. Fundus autofluorescence showed hypofluorescence consistent with these spots. Both the scotopic and photopic components of the full-field electroretinographies were non-detectable. Based on these clinical findings, this boy was suspected to have retinitis punctata albescens. Subsequent genetic testing using whole-exome sequencing revealed a novel homozygous variants in the *ARL*6/*BBS3* gene (NM_001278293.3:c.528G>A, (p.Trp176Ter)). A systemic examination by the pediatric department revealed that this boy had a history of a surgical excision of polydactyly on his left foot when he was born, and that he was mildly obese. There were no prominent intellectual or gonadal dysfunctions, no craniofacial or dental abnormalities, no congenital heart disease, and no hearing impairment. He was then clinically and genetically diagnosed with BBS.

**Conclusion and importance:**

In children with night blindness and progressive visual dysfunction, it is important for ophthalmologists to consult clinical geneticists and pediatricians to rule out the possibility of systemic diseases such as BBS.

## 1. Introduction

Bardet-Biedl syndrome (BBS: Online Mendelian Inheritance in Man; OMIM: 209900) is an autosomal recessive systemic disorder characterized by retinitis pigmentosa, polydactyly, obesity, cognitive impairments, renal abnormalities, and hypogonadism.^[1–4]^ The prevalence of BBS in Europe and the United States is estimated to be between 1/14,000 and 16,000 births,^[5,6]^ and it is known that the prevalence of BBS is higher in areas with many consanguineous marriages. BBS is a pleiotropic genetic disorder with significant interfamilial and intrafamilial variations.^[7]^ BBS can be molecularly classified into 22 types, as BBS22 (alternatively named IFT74) has been recently reported as a causative gene for BBS.^[8,9]^

The most common clinical feature of BBS is retinitis pigmentosa, but it differs from typical retinitis pigmentosa by being rapidly progressive, and the pigmentary changes which are characteristic of retinitis pigmentosa, do not appear until the disease is advanced. In BBS, the symptoms of night blindness generally appear by the age of 10 years, followed by progressive rod-cone dystrophy which often leads to legal blindness by 30-years-of-age.^[1–4,10,11]^ It is also known that the aforementioned 6 major systemic clinical features are not always present in patients with BBS, and some BBS cases have a so-called “asymptomatic” retinitis pigmentosa with only ocular symptoms.^[10,12–15]^ These variations make the diagnosis of BBS difficult.

The purpose of this study was to determine the characteristics of a Japanese boy with progressive night blindness and reduced vision who was initially diagnosed as retinitis punctata albescents based on clinical findings. Genetic analysis revealed the biallelic disease-causing variants in the *ARL6* and was diagnosed with BBS.

## 2. Case report

A 7-year-old boy visited a local eye clinic complaining of a progressive decrease in his visual acuity, and he was referred to the Mie University Hospital on suspicion of retinitis punctata albescens. He had a history of febrile convulsions, but there was no other systemic symptoms and signs. There was also no family history of eye diseases and no consanguineous marriage. His parents stated that he seemed to have difficulty in walking in the dark, and the symptoms had gotten worse over the years.

**Figure 1.**
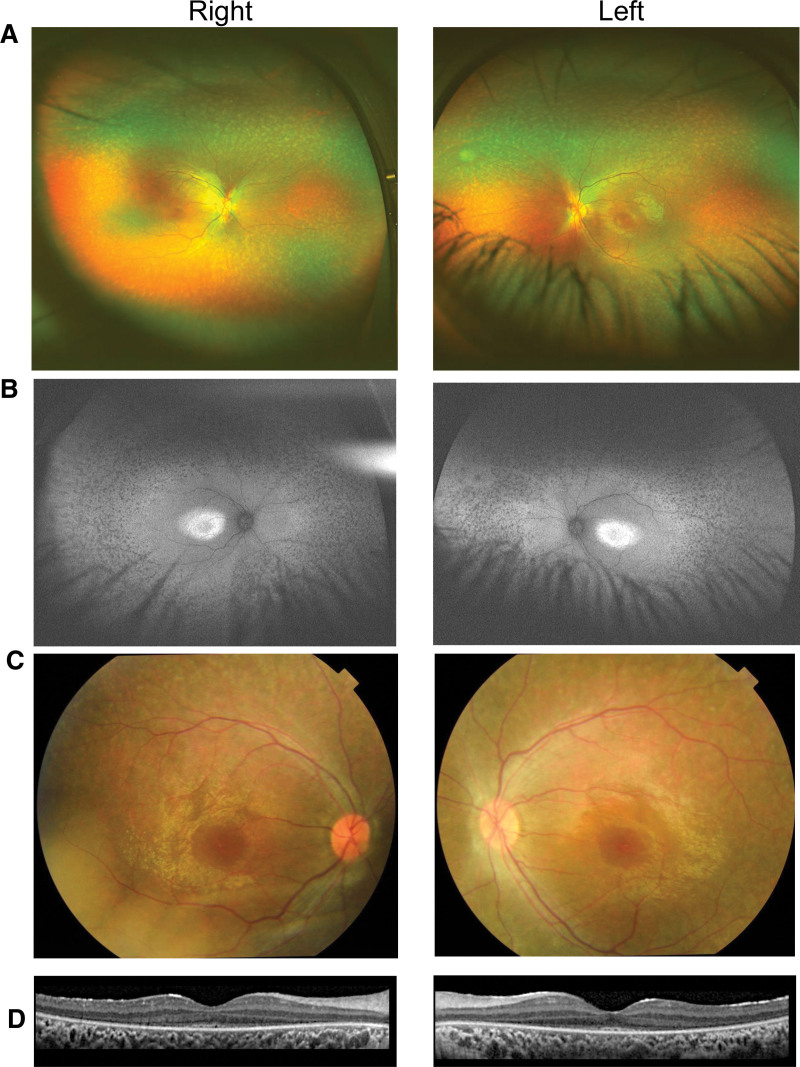
Fundus photographs, FAF images, and OCT images of a boy with BBS. (A) Fundus photographs showing numerous white spots over a wide area of the deep retina and retinal pigment epithelium of both eyes. (B) FAF images showing ring-shaped hyperfluorescence around the macula and multiple hypofluorescent spots which coincide with the white spots in the midperiphery of the fundus. (C) Fundus photographs showing the mottling of retinal pigmental epithelium sparing the central macula. (D) OCT images showing extensive degeneration of the outer retinal layers in both eyes, and the ellipsoid zone is barely visible and only in the central retina. BBS = Bardet-Biedl syndrome, FAF = fundus autofluorescence, OCT = optic coherence tomographic.

**Figure 2.**
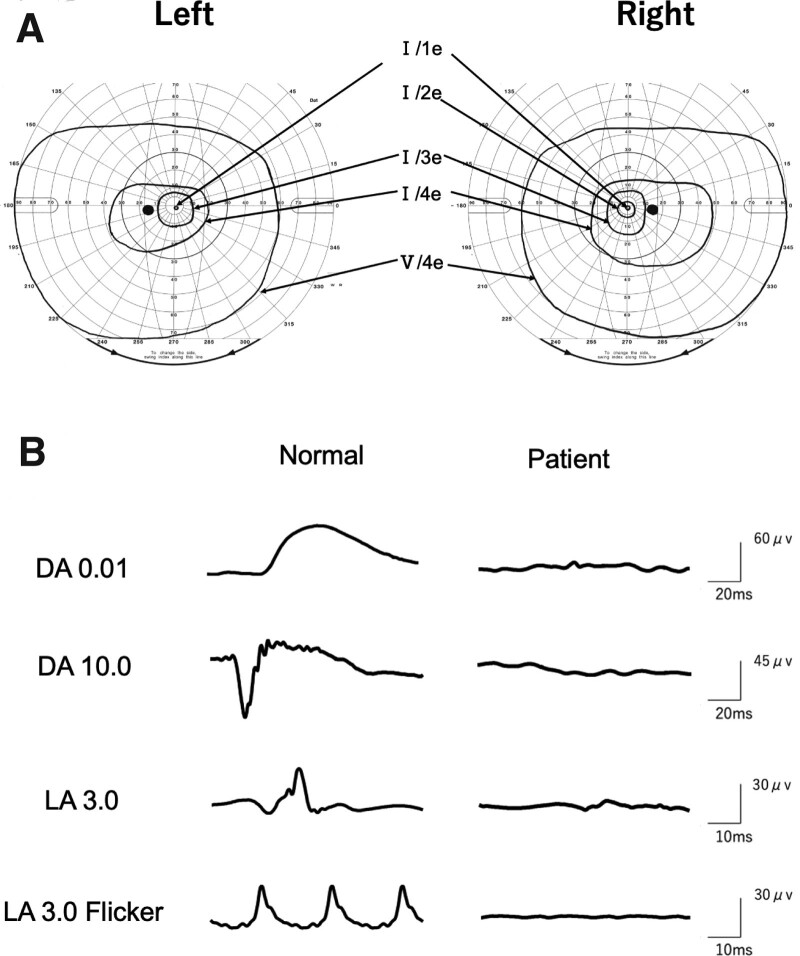
Goldman kinetic perimetry and ERGs of the patient. (A) The peripheral visual fields are intact with the Goldmann V-4 target, but a constriction of visual field was clearly detected with the I-4e to I-1e targets in both eyes. (B) Full-field ERGs of right eye recorded with the RET*eval* system. The scotopic ERGs were non-recordable and photopic ERGs were severely reduced. ERGs = electroretinograms.

His best-corrected visual acuity in decimal was 0.5 with a correction of +2.75DS = −3.00DC Ax 180° in the right eye and 0.4 with +3.00DS = −3.00DC Ax 170° in the left eye. The anterior segments were normal. Fundus examination showed numerous white spots over a wide area of the deep retina and retinal pigment epithelium in both eyes (Fig. 1A). An attenuation of the blood vessels was seen only in the peripheral retina. A waxy pallor of the optic disc was not apparent. Fundus autofluorescence demonstrated a ring-shaped hyperfluorescence around the macula and multiple hypofluorescent spots which coincided with white spots in the midperiphery of the fundus (Fig. 1B). Optical coherence tomography showed extensive degeneration of the outer retinal layers in both eyes, and the ellipsoid zone was barely visible only in the central retina (Fig. 1C). The peripheral visual fields with the Goldmann V-4 target were intact, but a constriction of visual field was clearly detected with the I-4e to I-1e targets in both eyes (Fig. 2A). Full-field electroretinographies were recorded according to the ISCEV guideline^[16]^ using the RET*eval* system^[17]^ and were almost non-detectable for both the rod and cone components (Fig. 2B). Based on these findings, we diagnosed the boy with retinitis punctata albescens.

We then performed genetic testing for a genetic diagnosis. After the approval of the Ethics Committee of Mie University Hospital (approval number: Mie-2429), genomic DNA was extracted from leucocytes in the venous blood samples. Whole-exome sequencing was performed with a targeted analysis of retinal disease-associated genes (http://sph.uth.edu/retnet/) according to published methods.^[2,18]^ The detected variants were filtered with an allelic frequency in the general Japanese population (<1% of the Human Genetic Variation Database, https://www.hgvd.genome.med.kyoto-u.ac.jp/) and the Japanese Multi Omics Reference Panel (jMorp, https://jmorp.megabank.tohoku.ac.jp/202001/downloads/legacy/), and the general population of gnomAD database (https://gnomad.broadinstitute.org/). The pathogenicity classification of all detected variants was performed based on the guidelines of the American College of Medical Genetics and Genomics.^[19]^

After filtering the whole-exome sequencing data, 12 variants were investigated based on the patient’s phenotype and inheritance pattern. Eventually, a novel homozygous nonsense variant {NM_001278293.3: c.528G>A, (p.Trp176Ter)} in the *ARL6* gene was identified. This variant is located in exon 7 of all 8 exons (position 49 of 56) and this variant is predicted to remove less than 10% of the ARL6/BBS3 protein. Given the presence of disease-causing variants located in the downstream of this variant on a previous publication {NM_001278293.3(ARL6/BBS3): c.535G>A, p.(Asp179Asn), an effect on the donor splice site confirmed by mRNA analysis} and the public database {ClinVar: NM_001278293.3(ARL6/BBS3): c.506del (p.Gly169fs), a putative truncation escaping nonsense mediated mRNA decay}, the pathogenicity of the variant (c.528G>A, (p.Trp176Ter)) was suggested. According to the American College of Medical Genetics and Genomics guideline,^[19,20]^ the variant was classified as “variant of uncertain significant” with some pathogenic evidence: PVS1 (moderate), and PM2. Out expert team, including clinical geneticists, ophthalmologist, and bioinformatician, decided the possible pathogenicity of the detected variant (c.528G>A, (p.Trp176Ter)) in the ARL6/BBS3 gene and the other rare variants of the retina-associated genes were excluded.

**Figure 3.**
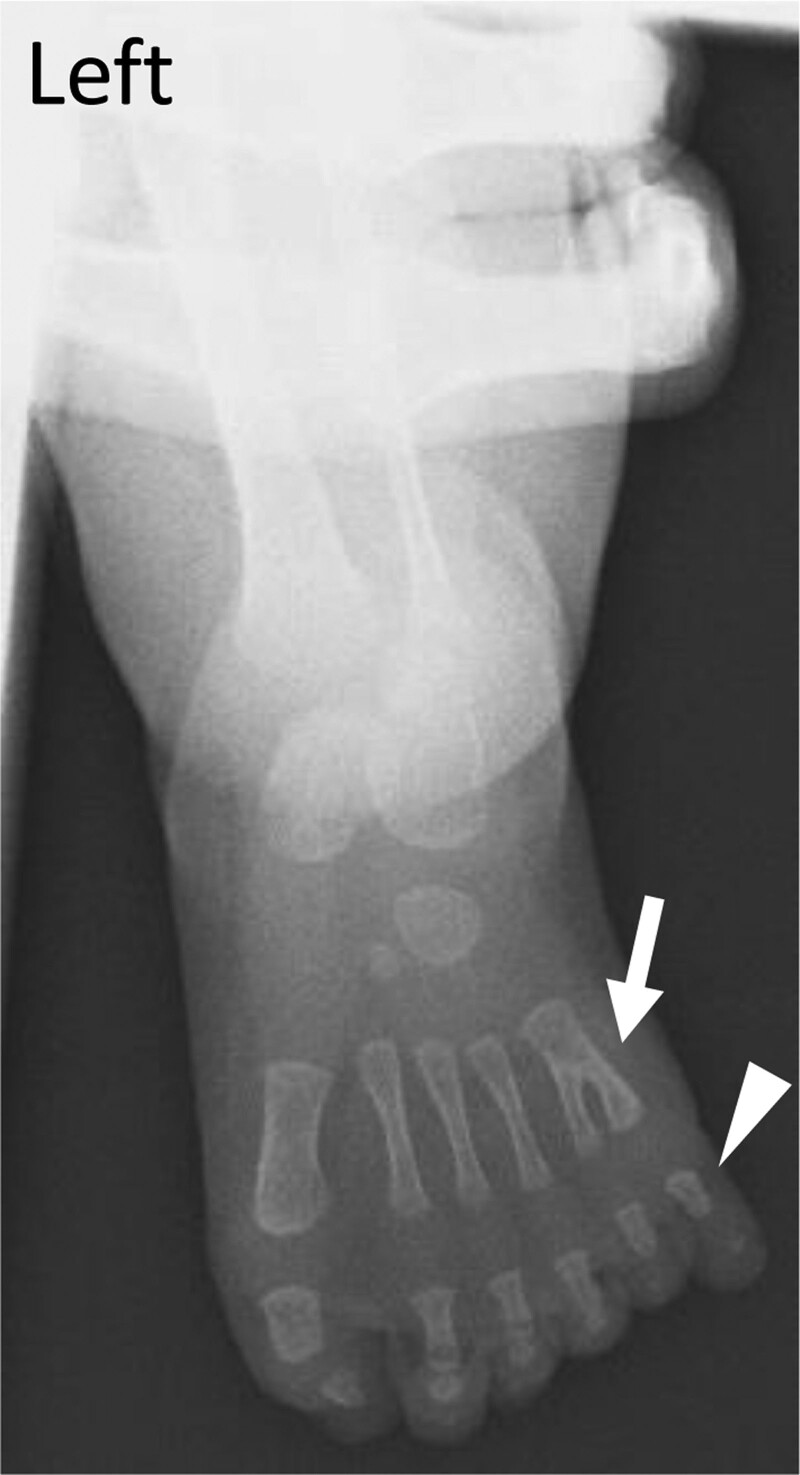
X-ray image of the left foot at birth showing polydactyly (arrowhead) and synpolydactyly (arrow) on his left foot.

Because pathogenic variants in the *ARL6* gene are known to cause BBS, we referred our boy to the pediatricians to perform systemic examinations. The results showed that he had mild obesity (height of 133.7 cm, weight of 48.5 kg, body-mass index 27.1 kg/m^2^, Laurel index of 202.9), although the obesity was not clear by body surface examinations. There were no obvious intellectual or gonadal dysfunctions, no craniofacial or dental abnormalities, no congenital heart disease, and no hearing impairment. A detailed interview with the parents on the child’s growth history showed that he had been diagnosed with polydactyly of his left foot at birth (Fig. 3) and had undergone excision. Finally, we diagnosed him to have BBS with retinitis pigmentosa, polydactyly, and mild obesity as the phenotype.

## 3. Discussion

We have presented the ocular and systemic findings in a Japanese boy with BBS caused by a novel homozygous nonsense *ARL6/BBS3* variant. This boy was initially diagnosed with retinitis punctata albescens based on the clinical findings including the multiple white spots, progressive night blindness, photoreceptor degeneration, and non-detectable electroretinographies. However, genetic testing revealed a homozygous truncating variant of the *ARL6*/*BBS3* gene {c.528G>A, (p.Trp176Ter)}, which was thought to be potentially disease-causing given the presence of pathogenic truncating variants located in the 3’ tail of the ARL/BBS3 gene. Then, we consulted with pediatricians and obtained a more detailed history of our patient, mild obesity and polydactyly were revealed. Three major findings and 1 minor finding of the BBS diagnostic criteria,^[6]^ as well as the results of genetic testing leading to the final diagnosis of BBS. As best we know, this is the first report of BBS caused by a homozygous nonsense variant in the *ARL6*/*BBS3* gene in Japan.

Among BBS, BBS1 and BBS10 being the most common, and BBS3 being relatively rare accounting for only about 3% of all BBS disease-causing variants.^[3]^ Disease-causing variants in *ARL6/BBS3* was first identified as the cause of BBS in a Bedouin tribe in which many consanguineous marriages are traditionally made.^[21]^ BBS with disease-causing *ARL6/BBS3* variants is mainly reported in the Middle East^[22–25]^ and La Réunion Island populations.^[26]^ The disease-causing *ARL6/BBS3* variants have been reported to account for 18% of all cases of BBS in India.^[27]^

The number of reported cases of BBS in Japan is low (Table 1).^[28–35]^ The causative genes reported so far are *BBS2*, *BBS5*, *BBS6*, *BBS7*, *BBS8*, and *BBS10*, and all but one of the cases were in non-consanguineous marriages (Table 1). The incidence of retinal degeneration, obesity, polydactyly, intellectual disability, hypogonadism, and renal dysfunction tended to be similar to those of patients in the reports from other countries.^[36]^ Polydactyly is more frequent in BBS, and polydactyly was observed in 80% of the cases in Japan. Although polydactyly is a clinical feature that is easy to identify, it is often treated very early in life, and it is common for parents not to report a history of polydactyly because of the negative associations of monogenic/Mendelian disorders. In fact, in our case, the parents did not voluntarily report the polydactyly. Thus, in cases of progressive night blindness in childhood, it is important for ophthalmologists to inquire about the systemic abnormalities especially polydactyly.

**Table 1:** Phenotypic and genotypic characteristics of previously reported cases with BBS in Japan.

Case	Age	Sex	CM	RP	CD	OB	PD	RA	HG	HGVS (cDNA)	HGVS (protein)	Reported genotype	Mutation type	Zygosity	Ref.
1	33	F	+	+	+	+	-	+	N/A	N/A	N/A	N/A	N/A	N/A	^[28]^
2	25	F	N/A	+	+	+	+	+	+	N/A	N/A	N/A	N/A	N/A	^[29]^
3	20	M	-	+	+	+	-	-	+	c.265C>T	p.Arg89Ter	*BBS5*	Nonsense	Hetero	^[30,31]^
4	16	M	-	+	+	+	+	-	+	c 265C>T	p.Arg89Ter				
5	9	F	-	+	-	+	+	+	N/A	N/A	N/A	N/A	N/A	N/A	^[32]^
6	6	F	-	+	+	+	+	+	N/A	c.728G>A	p.Cys243Tyr	*BBS7*	Missense	Hetero	^[31]^
7	2	M	-	N/A	+	+	+	-	+	c.1237C>T, c.1438C>T	p.Arg413Ter	*BBS2*	Missense	Hetero	^[31]^
											p.Arg480Ter				
8	22	F	-	+	-	+	+	-	N/A	c.98G>A, c.2125A>T	p.Gly33Glu, p.Arg709Ter	*BBS10*	Nonsense	Hetero	^[33]^
9	8	M	-	+	-	+	+	+	+	c.226C>T, c.308_309insTA	p.Gln76Ter, p.Gln104TyrfsTer63	*BBS8*	Nonsense/ frameshift	Hetero	^[34]^
10	7	F	-	+	-	+	+	-	-	c.589G>T	p.Gly197Ter	*BBS6*	Nonsense	Homo	^[35]^
11	7	M	-	+	-	+	+	-	-	c.528G>A	p.Trp176Ter	*BBS3*	Nonsense	Homo	present case

BBS, Bardet-Biedl syndrome, CD = cognitive disorder, CM = consanguineous marriage, F = female, HG = hypogonadism, M = male, NA = not available, OB = obesity, PD = polydactyly, RA = renal anomalies, RP = retinitis pigmentosa.

Among the 6 clinical features of BBS, retinitis pigmentosa is the most frequent at 90% to 100%^[36]^ and about 20% were retinitis pigmentosa sine pigmento.^[37]^ Although there are no reports on whether the ocular findings differ according to the genotype, a paper reporting the fundus findings of 12 patients between the ages of 4 and 17 years diagnosed with BBS3 reported that they had moderate myopia and had retinitis pigmentosa sine pigmento.^[38]^

In our case, retinitis punctata albescens was initially suspected. One question then arises as to whether such multiple white spots in the mid-peripheral retina are specific to BBS3. It is known that retinitis punctata albescens-like lesions can be often seen in various inherited retinal disorders,^[39]^ and 11 cases with retinitis punctata albescens-like lesions were reported in 2 BBS families in northern Canada.^[37]^ In that report, it was stated that the white spot lesions were found in the midperiphery in the early stages of retinal degeneration with mild loss of vision and minor macular abnormalities on ophthalmoscopy.^[5]^ Considering that retinitis punctata albescens-like lesions have also been reported at the early stages of typical retinitis pigmentosa,^[40]^ the multiple white spot lesions seen in our case was thought to indicate a rapidly progressive photoreceptor degeneration seen in the retina of childhood of BBS patients.

Twenty-five disease-causing variants of *ARL6/BBS3* have been reported: nonsense = 2, missense = 13, splice-site = 4, small-insertion = 1, and large deletions = 5 (Table 2). Of these, 18 variants developed BBS: nonsense = 2/2, missense = 9/13, splice-site = 2/4, small insertion = 0/1, and gross deletion = 5/5 (Table 2). Of these, the ones that develop non-syndromic ocular phenotype such as retinitis pigmentosa and retinal degeneration alone were 6 variants: missense = 4/13, splice-site = 1/4, and small insertion = 1/1 (Table 2). Among the BBS cases in Japan, 6 of the 7 cases for which genetic analysis was performed had nonsense variants (Table 1). These facts indicate that truncating variants with loss of total protein have a high probability of developing syndromic BBS, that is, retinal degeneration with several systemic disorders.

**Table 2:** A list of reported disease-causing ARL6/BBS3 variants.

Type	HGVS (cDNA)	HGVS (protein)	Reported phenotype	Impact of variants	Ref.
Missense	c.68T>C	p. Leu23Pro	BBS	NA	Jaffal et al^[25]^
	c.92C>G	p. Thr31Arg	BBS	Abrogate GTP binding	Fan et al,^[41]^ Kobayashi et al,^[42]^ Zaghloul et al^[43]^
	c.92C>T	p. Thr31Met	BBS	Abrogate GTP binding	
	c.266C>T	p. Ala89Val	RP only	Altering highly conserved amino acid	Aldahmesh et al,^[22]^ Pretorius et al^[44]^
	c.272T>C	p. Ile91Thr	BBS	Change the GTPase activation site	Sathya Priya et al,^[45]^ Chandrasekar et al^[27]^
	c.281T>C	p. Ile94Thr	BBS	NA	Khan et al,^[23]^ Carss et al,^[46]^ Kim et al^[47]^
	c.302G>T	p. Arg101Ile	BBS	Change the GTPase activation site	Sathya Priya et al^[45]^
	c.361C>T	p. Arg121Cys	Cone-rod dystrophy only	NA	Liu et al^[48]^
	c.362G>A	p. Arg121His	RP & rod-cone dystrophy only	NA	Patel et al,^[49]^ Abouelhoda et al^[50]^
	c.431C>T	p. Ser144Phe	BBS	NA	Abu Safieh et al,^[51]^ Maddirevula et al^[52]^
	c.506G>C	p. Gly169Ala	BBS	NA	Fan et al,^[41]^ Kobayashi et al,^[42]^ Zaghloul et al^[43]^
	c.509T>G	p. Leu170Trp	BBS	NA	
	c.529C>T	p. Leu177Phe	RP	NA	Gao et al^[53]^
Nonsense	c.4G>T	p. Gly2Ter	BBS	Stop gained, an entire loss of protein	Pereiro et al,^[54]^ Álvarez-Satta et al^[55]^
	c.364C>T	p. Arg122Ter	BBS	Stop gained, an entire loss of protein	Chiang et al,^[56]^ Xiong et al,^[57]^ Sharon et al,^[58]^
Splice site alteration	c.185+1G>A	NA	RP	NA	Neveling et al^[59]^
	c.479+3A>C	NA	Nephronophthisis only	NA	Braun et al^[60]^
	c.534A>G	NA	BBS	Skipping of exon 8	Maria et al^[24]^
	c.535G>A	p. Asp179Asn	BBS	Skipping of exon 8	Redin et al,^[61]^ Gouronc et al^[26]^
Small insertions	c.373dupA	p.Ile125AsnfsTer7	RP only	An entire loss of protein	Zenteno et al^[62]^
Gross deletion	c.732+1952_899-3806del4139	NA	BBS	Deletion of exon 8	Abu Safieh et al^[51]^
	c.123+1131del53985	NA	BBS	Deletion of intron 3-extending beyond the end of the gene	Chen et al^[63]^
	c.(?_-30)_(123+?)del	NA	BBS	Deletion of exons 1-3	Redin et al^[61]^
	NA	NA	BBS	Deletion of exons 4-9	Lindstrand et al^[64]^
	c.480-1710_535+2343del4109	p. Cys160Ter	BBS	Deletion of exon 8	Lindstrand et al^[64]^

BBS = Bardet-Biedl syndrome, RP = retinitis pigmentosa, NA = not available.

The ARL6/BBS3 c.528G>A, variant detected in our case is one in which tryptophan is replaced by a stop codon at 176 (p.Trp176Ter). This truncating variant located in exon 7 could escape nonsense-mediated mRNA decay; however, the presence of pathogenic truncating variants located downstream of the detected variant supports the potential pathogenicity of the detected variant in our case. The prevalent variant {c.535G>A, p.(Asp179Asn)} found in the La Réunion Island has been well-investigated, and the reported cases illustrate a severe clinical effect, including cognitive impairments of the variant that affected the donor splice site leading to the total skipping of exon 828 (Table 2).^[26]^ On the other hand, the variant {c.528G>A, (p.Trp176Ter)} detected in our case could not lead to an entire loss of the protein but create a truncated protein without the tail of exon 7 and the entire part of exon 8. Such a non-null change can be supportive in explaining the relatively milder phenotype of our case without cognitive impairments. Given the severe cases with the prevalent variant {c.535G>A, p.(Asp179Asn)} found in the La Réunion Island, the underlying mechanism of this variation for the phenotypic severity in a subset of ARL6/BBS3 cases with truncating/exon skipping variants in the 3’ side of the ARL6/BBS3 gene has been uncertain. Further analyses, including investigation of RNA/protein, could elucidate the disease mechanisms of the ARL6/BBS3-associated diseases.

In conclusion, detailed clinical and molecular findings were documented in a BBS case caused by a novel homozygous *ARL6/BBS3* variant {c.528G>A, (p.Trp176Ter)}. Notably in areas where the prevalence of BBS is low, ophthalmologists have few opportunities to encounter the disease, and the diagnosis of BBS is often difficult, especially when systemic complications are mild. In the case of retinitis pigmentosa which presents in childhood and progresses rapidly, it is necessary to conduct a detailed interview of the parents on the systemic and ocular condition/history of the patient, and to collaborate with clinical geneticists and pediatricians to ascertain the presence of other systemic features of BBS.

## Acknowledgements

We thank Professor Emeritus Duco I. Hamasaki of the Bascom Palmer Eye Institute of the University of Miami (Miami, FL, USA) for critical discussion and final manuscript revisions.

## Author contributions

KM, KK, KF, and MK wrote the main manuscript. TS, HA, SU, KF, and KT collected the data. All authors reviewed the manuscript.

**Conceptualization:** Kumiko Kato, Kazushige Tsunoda.

**Data curation:** Tadasu Sugita, Iichiro Sugita, Ayako Hattori, Shinji Saitoh, Shinji Ueno, Takeshi Iwata, Mineo Kondo.

**Formal analysis:** Kaoru Fujinami.

**Writing – original draft:** Keitaro Mizumoto.

**Writing – review & editing:** Kumiko Kato, Kaoru Fujinami, Mineo Kondo.
